# The Challenges of Public Health, Social Work, and Psychological Counselling Services in South Korea: The Issues of Limited Support and Resource

**DOI:** 10.3390/ijerph17082771

**Published:** 2020-04-17

**Authors:** Luis Miguel Dos Santos

**Affiliations:** Woosong Language Institute, Woosong University, Daejeon 34514, Korea; luismigueldossantos@yahoo.com or luisdossantos@woosong.org; Tel.: +82-010-3066-7818

**Keywords:** human resource shortage, mental counselling, psychological counselling, public health, shortage, social cognitive, social work, workforce management

## Abstract

Public health, social work, and psychological counselling professions in South Korea are facing challenges of human resource shortage and shortage of professionals who can provide multilingual services. The purpose of this study was to explore and understand why public health, social work, and psychological counselling services degree graduates and professionals with multilingual skills in South Korea decide to leave their professional field to the hospitality and business industries, particularly for those who completed their initial training at one of the international universities. Based on the approach of the Social Cognitive Career Theory, individuals’ self-efficacy, outcome expectations, interests, and goals were examined and considered. The data were collected from 12 participants with the methodology of interpretative phenomenological analysis. The general inductive approach was employed to categorize the themes for reporting. The results indicated that public health, social work, and psychological counselling services-related positions are not available, modelling from peers, and lack of career development skills are the primary difficulties of public health, social work, and psychological counselling services graduates. The completion of this study provides clear recommendations to educators, policymakers, school leaders, human resource planners, and university administrators to improve their curricula and school counselling for public health, social work, and psychological counselling services graduates and the next generation.

## 1. Introduction

### 1.1. Background of the Study: The Current Public Health, Social Work, and Psychological Counselling Services in South Korea

The shortage of health and social caring professionals is significant in many regions and cities, including those in South Korea. Public health, social work, and psychological counselling services are some of the foundational services [1] that can help social minorities, the elderly, refugees, vulnerable people, female residents, and even sexual minorities [2] to overcome some social, cultural, and financial difficulties in society [3]. Although the South Korean government has established some governmental agencies and foreign resident support centers with multilingual services, such centers cannot offer long-term, gradual, and follow-up services to some minority groups in the community [4]. Nowadays, nearly a million foreign residents are living in South Korea for various reasons, such as education, family reunion, work, and even giving political support [5]. However, there are only a few organizations that can provide public health, social work, and psychological counselling services to these minority groups. 

As a result, after completing their secondary school qualifications, many students decide to pursue undergraduate degrees, graduate degrees, and initial licenses in the fields of public health, social work, and psychological counselling services [6] at colleges and universities outside of South Korea with multilingual and inter-cultural training. Besides joining government agencies, large non-governmental organizations (NGOs), and non-profit organizations (NPOs), establishing their start-up NPOs may provide targeted services to particular groups of people in their specialization (e.g., refugee service, girls’ rights in rural communities, orphanage for abandoned girls, etc.) [7]. However, such NGOs and NPOs are rarely found in the current South Korean environment.

Moreover, although South Korea is an international region with multi-disciplinary services, industries, and business options, the South Korean government tends to invest resources in the hospitality, tourism, and service management sectors [8]. Hotels, restaurants, entertainment, and similar industries employ many local and international residents. Although there are no official statistics regarding human resource management and occupational background, many local people and residents work in these industries. Based on the current employment trends of the region, many graduates with majors other than business and hospitality may not be able to work in the industries of their backgrounds, particularly for South Korean graduates who completed their education at an international university. Such unhealthy environments may limit the diversification potential of South Korea. 

### 1.2. Purpose of the Study

The purpose of this study was to explore and understand the factors contributing to the career decisions and decision-making processes of recent public health, social work, and psychological counselling services graduates in South Korea, particularly for those with degrees from overseas universities who are working in industries other than public health, social work, and psychological counselling services. Based on the approach of Social Cognitive Career Theory (SCCT) [9], individuals’ self-efficacy [10,11,12], outcome expectations, interests, and goals [13,14] were considered and examined. 

First, studies [15] indicate that recent graduates tend to enter industries corresponding to their academic majors and personal interests. Although financial consideration is a critical element in career development, public health, social work, and psychological counselling services professionals tend to consider personal interests and outcomes developments as their priorities [16,17,18]. One report [19] indicates that public health, social work, and psychological counselling services graduates in western societies usually start their centers or join NPOs after graduation. However, recent public health, social work, and psychological counselling services graduates in South Korea are not interested in starting businesses or NPOs. Therefore, the researcher aimed to understand the reasons for the career decisions and decision-making processes of recent public health, social work, and psychological counselling services graduates [20].

Second, although the South Korean government has financially and administratively supported many types of start-up NPOs and NGOs, unlike other professionals, individuals in the fields of public health, social work, and psychological counselling services have no interest in establishing businesses or NPOs of any kind in the public health, social work, and psychological counselling services industries. It is essential to understand the underlying reasons for this [21]. 

Third, one study [19] indicates that public health, social work, and psychological counselling services professionals tend to start their centers or NPOs after graduation, particularly with support from government agencies. Although the South Korean government has established planning for supporting such endeavors, public health, social work, and psychological counselling services professionals tend to give up and leave the public health, social work, and psychological counselling services industries to further develop their careers in other areas. The study aimed to explore this unique behaviour [22]. 

Fourth, career development theories have been based in large part on studies with university students, career changers, and working adults. Underrepresented populations have different career desires and face difficulties concerning their social status, academic majors, networking, and career development [14,16]. However, there are only a few research articles concerning the career pathways and development of public health, social work, and psychological counselling services graduates, particularly in South Korea. Most research articles in public health, social work, and psychological counselling services examine how to incorporate public health, social work, and psychological counselling services into the curriculum, and how to enhance practices for patients. This is because public health, social work, and psychological counselling services educators tend not to feel strongly about the further development of graduates. Therefore, a large gap in career counselling and development is found for public health, social work, and psychological counselling services graduates. An emerging area of research focusing on career development has concentrated primarily on hospitality workers’ perspectives and human resources shortages [23]. Graduates with academic majors other than business-related subjects represent a significant proportion of the region’s human resources, particularly graduates with public health, social work, and psychological counselling services degrees from overseas. Yet, questions remain about the career perspectives and career planning of these groups of residents [1]. 

### 1.3. Theoretical Framework

This study employed SCCT [9,13,14] to explore the career decisions and decision-making processes of recent public health, social work, and psychological counselling services graduates, particularly those working in industries other than public health, social work, and psychological counselling services. SCCT [24,25,26] is a famous theoretical framework for vocational and academic predictors of interests, career choice options, and performance. However, only a few studies explore the career and vocational perspectives of residents in South Korea. 

More importantly, research on the views and behaviors of public health, social work, and psychological counselling services professionals are absent. Therefore, the result of this study may increase the social attention paid to public health, social work, and psychological counselling services professionals. The outcome of this study may lead to recommendations for policymakers, human resource professionals, secondary school staff, parents, career counsellors, and university administrators in planning career developmental plans for the next generation. 

SCCT [9,14] was developed to understand and explore career perspectives and intentions. It aims to explain, describe, and explore academic and vocational decisions and performance, and the persistence of educational and vocational goals. This framework examines how people apply personal factors in the occupational and career development procedures and how personal elements increase, decrease, or otherwise impact personal agency. SCCT also indicates the significance of Social Cognitive Theory and triadic reciprocal causality [23]. Triadic reciprocal causality is an inter-influential point which impacts the connections, interactions, and significances among people, behaviors, and environmental factors. Activities, thinking, and behaviors of people are not the results of inter-connected events between people and environmental and social elements; instead, behaviors act as inter-connected elements by impacting and influencing results, thereby impacting the personal, emotional, intrapersonal, and further movements, activities, decisions, and behaviors of people [9,14,17,27,28]. 

Given SCCT’s notions about how people, behaviors, and environmental elements could impact and influence the career decisions and decision-making processes of people, in this case, recent public health, social work, and psychological counselling services graduates, this study explored the factors that may be related to their career decisions and decision-making processes. The SCCT works as an effective framework when seeking to address the gaps in the current debate relevant to making the career decision to work in an industry other than that relevant to one’s educational goals and interests. 

## 2. Materials and Methods

Based on the structure of the SCCT [9,14] and the significances, one research question guided the direction, which was: Why do recent South Korean public health, social work, and psychological counselling services degree graduates with overseas degrees decide to work in the fields other than public health, social work, and psychological counselling services? The purpose of this chapter was to outline the research methods, including research design, participants, instrumentation, data collection procedures, and data analysis procedures. 

The researcher decided to employ the Interpretative Phenomenological Analysis (IPA) in understanding how lived stories and in-depth personal understanding associated with social, education, financial, and personal goals influenced the career decision and decision-making process of recent public health, social work, and psychological counselling services graduates who are working in the industries other than public health, social work, and psychological counselling services [29,30]. Previous Interpretative Phenomenological Analysis studies have shown how individuals and participants make sense of their personal and social world and experience. As the aims of this study tended to collect in-depth understanding and rich lived stories from the participants, the Interpretative Phenomenological Analysis was appropriate as the methodology. The researcher employed the qualitative research method with purposive sampling for recruitment. 

### 2.1. Participation

The sample involved 12 participants. First, the Interpretative Phenomenological Analysis seeks to understand the in-depth understanding and lived stories. Each represents a lived story that may be extremely meaningful. The data collection and analysis may highly satisfy. Second, the general phenomenological analysis may recruit at least ten participants and up to 200 participants. Large sample size may eliminate and cover up the rich lived stories from each participant. Also, as this study primarily focuses on the in-depth understanding of career development for South Korean residents who completed their public health, social work, and psychological counselling services degree overseas, a small sample size was more reasonable [31]. 

A purposive sampling strategy [31] was employed to invite 12 students who graduated overseas. Participants’ demographic information, such as name, age, gender, year of experience, educational background, etc. was collected. However, the identifications of participants were pseudonym to protect the privacy. 

The participants of this study satisfied the following requirements,

- Currently working in a field other than public health, social work, and psychological counselling services;
- Graduated no more than three years;
- Completed their academic degree in the field of public health, social work, and psychological counselling services from an international university outside South Korea;
- At least 18 years old;
- Non-vulnerable person;

The researcher contacted each potential participant by email invitation. The invitation letter provided the information including the nature, objective, aim, and methodology, requirement of participants, and purpose of the study with a declaration about their voluntary participation or non-participation. If the participant agreed with the research, the participant responded to the email for further actions. For detail about the participants’ information, please refer to Table 1.

### 2.2. Data Collection

The researcher was the primary tool for data collection and analysis. Participants usually shared personal background, lived stories, and career decision with people who they can believe. To establish a solid relationship, the researcher designed two rounds of a semi-structured and one-on-one interviews. The member checking interviews were conducted after the completion of data analysis. Therefore, both met each other for at least three interview sessions in total. Each semi-structured interview was between 60–90 min. The member checking interview was hosted for about 30 min. Pseudonyms were assigned to shadow their privacy [32].

### 2.3. Data Analysis

Themes, patterns, and groups that were categorized during the interview sessions were individually mapped. The general inductive approach was employed to analyze. The general inductive approach allowed researchers to explore the interview transcripts. First, the researcher followed the general inductive approach to reduce the large-size interview transcripts into the first-level themes by employing the open-coding strategy based on the direction of the grounded theory approach. Due to the detailed and in-depth interview sessions, the researcher captured 700 pages of interview transcripts. After the open-coding strategy, the researcher categorized 19 themes and 28 subthemes for the first-level reporting [33]. 

Second, based on the first-level themes and subthemes, the researcher reduced the interview transcripts into the second-level themes and subthemes with the axial-coding strategy. Several qualitative researchers [31,33,34] have indicated that the axial-coding strategy allows the researcher to narrow down the first-level themes and subthemes based on the research question. Therefore, three themes and four subthemes were categorized.

### 2.4. Human Protection and Ethical Consideration

The study involved 12 participants in South Korea. The protection of human subjects was important to this study, particularly given the study’s focus. Therefore, the researcher made every effort to protect the identities of all those involved, allowing them to remain anonymous to any parties in society. In the report, each person was identified solely by their role. 

All subjects gave their informed consent for inclusion before they participated in the study. The study was conducted in accordance with the Declaration of Helsinki, and the protocol was approved by the Ethics Committee of Social Caring Center (Summer/2019). 

## 3. Results

During the interview, the participants answered the same general open-ended questions about their educational background, ideas regarding public health, social work, and psychological counselling services in the current South Korean environment, expectations to do with their degrees, personal goals, the social expectations of the public health, social work, and psychological counselling services professions, governmental policy toward the public health, social work, and psychological counselling services industries, and so on. Although all had similar interests in the fields of public health, social work, and psychological counselling services, some of their shared experiences, lived stories, and financial considerations were not the same. The situation in South Korea is unique. As a small region with a large population, people’s lifestyles, family structures, conceptions, and understandings may have many common grounds while also having many differences due to geographic elements. To answer the research question in a structured order, this section is categorized into three themes and four subthemes based on the interview transcripts and information from the participants. Table 2 outlines the themes and subthemes of this study. 

### 3.1. Public Health, Social Work, and Psychological Counselling Services-Related Positions are not Available

The high percentage of hospitality employment reflects that most available positions are in a single industry. Professionals from other fields are less likely to expand their expertise in their own fields. In the current study, all participants were in the field of public health, social work, and psychological counselling services, where employment is less likely to be found in the business and non-profit sectors.

All participants had public health, social work, or psychological counselling services degrees from overseas institutions, but none of them were able to work in public health, social work, and psychological counselling services-related positions due to the shortage of opportunities. All except Participant#8 and Participant#9 believed the government did not emphasize the public health, social work, and psychological counselling services industry enough. They referred to the current public health, social work, and psychological counselling services environment as a “desert” to reflect the lack of support. Participant#9, who had a psychological counselling degree, but was working as a restaurant servant, asserted the dead-end nature of the counselling services environment, saying, “the hotels kill all other professionals. The tourism industries are the big dragon. No one in the city even supports psychological services.” Participant#2 echoed this negativity, saying, “I tried to ask the Government Department for support for the support of sexual minorities’ services for foreign residents in Seoul…not a hard request, but only negative news.” Participant#10 also expressed that there were no opportunities for recent graduates, saying,

*…for those who want to join the field, there are only two ways, either start your own non-profit or enter the government. Public health, social work, and psychological counselling are not easy jobs…in South Korea, even if I graduated from a top-tier university, there are no positions…it depends on internships and networking…at least I don’t have savings to start my own [organization]. Even if I do have millions, I cannot recruit all graduates…*

Participant#12’s academic background was similar to Participant#10’s (e.g., psychological counselling), but Participant#12 worked in the surveillance office due to his video-related skills (i.e., academic minor). Although Participant#12’s working position was related to video, the job specifications and responsibilities were not the same as for public health, social work, and psychological counselling services, as Participant#12 explained, saying, “Looking at the camera for potential cheating is not the same as making meaningful and enjoyable videos for the minorities in my country. I cannot say I entered into the public health, social work, and psychological counselling services, this is hospitality…” Participant#3 asserted that many orphanages and religious churches are operating in South Korea. Although a few places recruit people without networking and connections, as Participant#3 said, “South Korea is an international region…but the governmental agencies and large-size non-profit organizational heads recruit their own team members…for us…there are no openings.” Participant#5 and Participant#6 were hotel front line attendants at the same company. Both used the statement “no future, no dream for our public health, social work, and psychological counselling services career” as an ironic pun on the hotel’s slogan. Participant#7 somehow used his professional skill to make fun of his current position saying,

*I wish I could leave this meaningless position in valet parking. But I am glad that I can laugh in front of all customers all the time. At least I learnt social work skills. Even if I don’t want to work, I must work for basic living.*

Participant#4 continued to seek openings as a professional in the fields of public health, social work, and psychological counselling services in the near future, but his expectation of potential opportunities in public health, social work, and psychological counselling services was weak, as he explained, saying,

*The government said there are rooms to open our center. But it has been four years. I want to go back to public health, social work, and psychological counselling services. But can I come back? I am afraid six years later I will not have the courage to leave my position in this hotel.*

Participant#8 did not express much negative thinking about her position, but believed professionals in the field of public health, social work, and psychological counselling services should able to take hardship, saying,

*I understood there were no opportunities once I graduated. This was my own choice…I cannot work as a professional in the field of public health, social work, and psychological counselling services now. But I can enjoy chatting with my customers in the hotel. I can help my customers and other co-workers at my workplace…But this is very stupid…I have my professional skills in the field of public health, social work, and psychological counselling services…But I cannot use my multilingual skills and professional skills to help the correct minorities and people who are suffering from pain…*

Participant#11 worked in the marketing department. The department relied on Participant#11’s photographic and art therapy skills to put pictures in emails. She said, “my responsibilities are 20% related to my public health, social work, and psychological counselling services profession, particularly art therapy. All footnote pictures at the bottom of emails in my department were my art. But I am only responsible for typing and responding to emails between my company and customers.” Participant#1 was the only participant who was not working in the hospitality and hotel industry. Her viewpoint was slightly different from the others’, who were working in the hospitality industry, saying,

*…I enjoy working as an administrative assistant. I can coordinate some printing workshops with the center users and members. I don’t think I can open my center because I don’t understand how to operate a counselling center. I would rather use the money for my down payment…for my apartment and unit…perhaps my children in the future?*

All participants were working in industries other than public health, social work, and psychological counselling services. Recent graduates with public health, social work, and psychological counselling services degrees may apply their professional skills and abilities to other professional environments. For example, Participant#11 applied her photographic and art therapy skills in her marketing department and email designs. However, most of the participants worked in fields that were totally removed from public health, social work, and psychological counselling services. Many expressed negative comments due to the mismatching of career expectations and personal development. For example, Participant#3 worked as a ticket seller in a hotel’s box office. This mismatching may further create a high level of turnover due to dissatisfaction.

### 3.2. Modelling Peers

Starting a career pathway is not an easy step for recent public health, social work, and psychological counselling services graduates without much working experience or connections. First, unlike their counterparts in business schools or vocational training institutions, students in public health, social work, and psychological counselling services programs may not need to complete a business-oriented, practical internship for their professional year. Second, all participants completed their academic degree at a university overseas. Most were therefore unable to work in organizations outside the university setting without visa sponsorship. The visa requirement limited their opportunities to seek appropriate working experience during their academic career. Third, South Korean students do not have the right of abode overseas. Most had to leave their host countries within a certain period after graduation. Even if they had built up strong connections and good networking, they had to leave after graduation. Therefore, the absence of social and vocational connections within the professional field in South Korea was disadvantageous for this group of public health, social work, and psychological counselling services graduates.

#### 3.2.1. Modelling and Referral from Classmates

All participants worked in the hospitality industry except for Participant#1. One of the strongest reasons why graduates with public health, social work, and psychological counselling services degrees joined the hospitality industry was peer influence. All participants were originally from South Korea, where they completed their secondary education. Therefore, most of their peer connections and career recommendations were in South Korea. Participant#2, Participant#5, Participant#6, and Participant#8 said that they entered the hospitality industry due to suggestions from their secondary classmates. Participant#2 said,

*Several of my classmates studied hospitality and tourism management. They were able to seek their first full-time position right after their internships. Therefore, they referred me to their department supervisor and recruited me. I am so fortunate to have a full-time job after I came back.*

Participant#5 echoed a similar expression about referral from classmates, saying,

*In South Korean culture, references from others are key to finding opportunities. My classmates in the business administration program told their bosses to recruit me. I applied for many positions…but no responses…most of my friends were doing well in the hotel, so I wanted to join and try too.*

Participant#6 elaborated on peer influence and her first position in the hospitality industry, saying, “my secondary classmates are successful in the hotel. I seriously don’t think the government is going to support my center. To survive, I follow my classmates’ footsteps and make some money for living.” 

#### 3.2.2. Modelling and Referral from Cousins

Participant#4, Participant#7, and Participant#9 were influenced by their same-age cousins who were in the hospitality industry. Participant#4 entered the reservation department due to the peer influence from his female cousin in a similar position, saying, “my cousin is a reservation assistant supervisor and says the workload is okay. I keep writing poems and storybooks during the days off.” Participant#7’s cousin was promoted to a supervisor position about two years ago. Participant#7 was able to secure his position due to the referral of his cousin, saying,

*I sent out my applications and CV [curriculum vitae] at the beginning of my last year of university. No responses or interviews. I knew my cousin was working in the parking department, so I sought him out for help. I cannot say I like it, but I know I must survive.*

Participant#9 also sought her first position in a different field based on the referral from a cousin, saying,

*Public health, social work, and psychological counselling services are not a trend…the biggest companies are in France, the United Kingdom, Italy, the United States and other western countries. I understand the direction has been switched. I cannot wait for a lottery…after 15 months of unemployment, my cousin helped me to send out my CV to her department head in a hotel restaurant…*

It is important to note that modelling peers and classmates reflects the central element of Social Cognitive Career Theory. Scholars [9] have further advocated that modelling other people’s success stories may highly influence individuals’ career choices and behaviors. More than half of the participants switched their career direction from public health, social work, and psychological counselling services to hospitality due to the strong influence of their peers. These participants may be going against their own principles. However, peer influence changed their points of view about long-term and short-term career pathways.

### 3.3. Lack of Career Development Skills

Most public health, social work, and psychological counselling services programs do not provide vocational and career-oriented training and preparation for seeking opportunities in the business environment [21]. In fact, many organizations want to recruit business professionals to increase the image of their departments, particularly for marketing advertisements. However, public health, social work, and psychological counselling services graduates usually do not understand how to apply their professional and counselling skills in a business environment. Therefore, most public health, social work, and psychological counselling services graduates are unable to apply their professional skills in appropriate directions.

#### 3.3.1. Afraid to Start Own Centers and Non-Profit Organizations

Three participants expressed that they had planned to start their own centers or NPOs during university. However, these participants stated that they did not understand how to begin, maintain, promote, operate, and continue such endeavors. Therefore, after consideration, they terminated their plans. Participant#3 shared his experience of establishing a center, saying,

*During university, the only public health, social work, and psychological counselling services professionals that I could encounter were my lecturers. They were very successful social workers, counsellors, and health professionals. But I could not learn ideas from them about establishing my own center. Most of them never started their own centers, so how could they have taught me?*

Participant#1 expressed another idea about the absence of career development, saying,

*I don’t know how to attract residents and tourists to my center…if I start in a small community. The program curriculum does not have such courses…they only trained us as a professional service provider. But professional service providers also need money to survive.*

Participant#6 further emphasized the feelings from Participant#1 saying,

*I know how to promote sexual health, elderly service, youth service, and women’s issues…During the last year of university, we had to learn how to serve multi-cultural and social disabled people from countries with political unrest…but I wanted to start my center. But I somehow didn’t know how to start the center. One or two counselling professionals may operate many of the centers in the market. But these centers don’t hire outsiders. We have to start our own. But I didn’t have the business sense…*

#### 3.3.2. Lack of Interdisciplinary and Practical Skills

Based on the interviews, the researcher noted that almost all the participants did not understand how to apply their valuable skills in the practical and professional environment outside of the public health, social work, and psychological counselling services professions. In other words, most of the participants only had skills in their own public health, social work, and psychological counselling services subjects, and no other professional skills. Due to the absence of interdisciplinary and practical skills required from potential employers, they were unable to expand their horizons to the next stage. For example, Participant#2 expressed her hardship in seeking employment, saying,

*Many international hotels are using technology to design their art products. But I am still in the 70s. I know how to draw and paint in watercolors with art therapy. But how to use a computer to print and how to use the computer to assist…I don’t know.*

Participant#9 also applied for the wardrobe and linen department at a hotel. Participant#9 should have applicable skills in clothing, so such a position should have been appropriate. However, Participant#9 expressed that her skill was not transferable, saying,

*My skill is in counselling and visual counselling. I know how to use a different color. My interests are all about color, cutting, and fitting. But in the wardrobe and linen department, they mainly focus on washing the clothes. I never learned that at university. I wish I understood, but I don’t want to lie to the manager.*

Participant#4 should be a good writer and even speaker for documents and advertisements, but did not know how to apply this skill to a business environment, saying,

*…I applied for a newspaper journalist position. I wanted to write some articles for the forums. But they only recruited from the traditional section. I never studied, so I was refused an offer. For now, I apply for other contract writer and advertisement writer positions. Their requirements and expectations are not in my expertise…*

Participant#5 and Participant#7 both expressed the same sentiment, saying, “how can I apply my professional skills into a business environment…a good smile is okay.” 

All public health, social work, and psychological counselling services graduates should be experts in critical thinking and problem-solving. However, most were close-minded in other professional areas, particularly business. Public health, social work, and psychological counselling services elements are not hard to find in luxury hotels and shopping centers. Most expressed that their public health, social work, and psychological counselling services skills should apply in their particular direction. While some participants may have taken jobs in different industries due to money issues, resistance to applying their professional skills was also obvious.

## 4. Discussion

The purpose of this section is to discuss the themes shared by participants and implications for the career decisions and development of public health, social work, and psychological counselling services university graduates, using SCCT as a theoretical lens. Based on the idea of SCCT, personal factors (i.e., personal beliefs, biological elements), human behaviors, and external environmental elements (i.e., social movement, society) are the central elements influencing the career choices and selections of individuals [9,17,35]. However, based on the findings of this study, external environmental elements strongly impact career selection. 

First, the researcher sought to understand better why public health, social work, and psychological counselling services graduates with overseas degrees plan to work in an industry other than their academic major. Individuals usually do not select and enter careers in which their self-efficacy is lower than average [9]. However, the study discovered that all participants needed to enter an industry in which they had no experience. All sought public health, social work, and psychological counselling services-related employment opportunities but none of them were successful. Environmental elements strongly impacted their career decisions, more than human behaviors and personal factors. External environmental and ecological barriers in society can influence the career decision making of individuals [16]. One of the most significant contextual barriers to career decisions was the limited public health, social work, and psychological counselling services-related positions in the region. The researcher was interested in understanding whether the outcome expectations of participants would also influence their career decisions, as in another study [14]. Although outcome expectations were considered a lower priority in the current study, several of the participants applied part of their public health, social work, and psychological counselling services skills in their workplace. 

Second, it was surprising that almost all were working in the hospitality and service management industry, particularly the field of hotel management. Although there are also other industries, such as small business, government, transportation, and even finance, most decided to enter the hospitality and service management industry. Based on sharing from the participants, most were influenced by their social peers, classmates, family members, and the social situation. In line with SCCT [9,14], it was clear that modelling was one of the most important elements impacting individuals’ career decisions and development out of the external and environmental factors. For example, Participant#2 said that most of her friends and classmates were able to achieve promotion to a reasonable position after their graduation due to the growing hospitality and service management industry, saying “regardless of my major, as long as I am willing to work in service management, I can work and potentially…career promotion and advancement…” In the current situation, external and environmental factors highly influenced how individuals selected their careers and their career development, regardless of their university major and background [22]. 

Third, other evidence to supporting the importance of external and environmental factors came from the influence of families and cousins. Families’ and cousins’ recommendations are the strongest factors influencing individuals’ career decisions and development, particularly for individuals with a South Korean background. SCCT [9,14] asserts that individuals are influenced by external and environmental factors differently. In this case, individuals tended to be significantly influenced by external and environmental factors. For example, Participant#7 indicated that his cousin transferred his CV. Also, Participant#9 indicated that as there were no openings in her profession, she used her cousin’s connections to get her first full-time employment. SCCT argues that individuals’ social background, social consideration, and the current social environment always influence individuals’ career decisions and development [9,13,14]. In this case, although the participants gained essential skills and abilities from their university education and training, they tended to enter and start their career and its development based on external and environmental factors [21]. 

Fourth, some claimed that they did not have the practical skills and understanding to establish a center with limited resources [21]. Unlike in other countries and cities, recent graduates usually do not have enough resources. Therefore, graduates with limited resources usually cannot afford the operating costs. Furthermore, some explained that the university curriculum did not offer any training in establishing a center. Therefore, self-efficacy [36] and personal intention [24] were low. As a result, most were influenced by external and environmental factors as their surrounding society and environment always encouraged them to follow social expectations and trends [9,13,14]. 

In short, individuals’ career decisions and career development may be explored and described using SCCT [9,13,14] with personal factors (i.e., personal beliefs, biological elements), human behaviors, and external environmental elements (i.e., social movement, and society) as the key elements. In the case of public health, social work, and psychological counselling services university graduates, most were highly influenced by external and environmental factors due to South Korean cultural expectations and limited career opportunities. Unlike other countries and cities with reasonable rental fees, space, and career opportunities, South Korea does not provide additional support and opportunities for graduates to exercise their skills and practices with limited resources, regardless of their majors and skills, particularly for overseas graduates. Therefore, regardless of university majors and skills, most of the participants in this case expressed that their only employment opportunities after university graduation were in the hospitality and service management industry [1]. 

## 5. Limitations, Future Research Directions, and Conclusion

### 5.1. Limitations and Future Research Directions

Some may argue that the number of participants was too small. However, the in-depth and two-round interview sessions were used to overcome this limitation. First, this study employed the Interpretative Phenomenological Analysis methodology to capture in-depth understanding, lived stories, and sharing from the participants. The research study tended to capture how individuals made sense of and understood their social world. Thus, the results of this research study allowed a rich and detailed understanding of the background of the social problem. 

Second, some may argue that the background of the participants was limited to the fields of public health, social work, and psychological counselling services. Each research study needs to focus on a group with a focused direction. Therefore, future research studies and projects may expand the current research to additional groups of people, such as medical professionals. As social problems, limited career opportunities, and human resource shortages may influence all industries and businesses as a general problem in South Korea, larger and wider studies and projects would be beneficial to all residents and government leaders. 

Third, this research provides a blueprint to government leaders, human resource managers, school administrators, policymakers, university curriculum heads, and general residents for a background understanding of the shortages in the fields of public health, social work, and psychological counselling services in South Korea. This research study gathered feedback and sharing from a group of public health, social work, and psychological counselling services graduates who needed to enter other industries due to the limited career opportunities in the region. Such feedback and sharing should be an indicator to all government leaders to reconsider the region’s social policy and management for the coming fiscal years. Therefore, interested researchers and government leaders should take this research study as a starting point for redesigning the social policy regarding the public health, social work, and psychological counselling services industries and related small organizations and companies in the region. 

Fourth, countries, cities, and regions with similar situations may use this research to polish their current social policies and activities [1]. The situation in South Korea is extreme and hard to solve due to the chaotic management of the last few decades. Government leaders, policymakers, and human resource managers should have acted to improve the situation before this point. 

### 5.2. Conclusions

This study discovered three critical social situations in South Korea. First, South Korean society is unique to other similar countries and cities internationally. Unlike in other larger countries and cities, limited career opportunities, lands, and industries always prohibit skilled professionals from excelling in their areas of interest and skill based on their university majors and training. A large number of residents in South Korea are employees in the hospitality and service management industry. Although the government has established plans and agendas for additional industries and small businesses, most of the residents do not intend to start their businesses due to limited knowledge and environmental factors [1]. 

Second, the participants in this study tended to be influenced by external and environmental factors, in accordance with SCCT [9]. The results indicated that all had low levels of self-efficacy and confidence about starting their centers due to the social environment and expectations. Therefore, although most of the participants had the passion to start their own businesses in the fields of public health, social work, and psychological counselling services, most tended to enter the hospitality and service management industry based on social influences either from society or their peers. 

Third, South Korea should spend additional resources on and give more consideration to industries and small businesses other than the hospitality and service management industry. Although the revenue and tax incomes of South Korea highly rely on the hospitality industry, the government should not neglect the development and promotion of other industries, such as public health, social work, and psychological counselling services, and even medical tourism [8]. 

## Figures and Tables

**Table 1:** Biography of the participants.

Name	Gender	Age	Major	Current Position
Participant#1	F	24	Public Health	Administrative Assistant
Participant#2	F	23	Social Work	Administrative Assistant
Participant#3	M	23	Health Promotion	Ticket Seller
Participant#4	M	25	Social Work	Reservation Attendant
Participant#5	M	26	Social Work and Mental Counselling	Hotel Front Office Attendant
Participant#6	F	23	Mental Counselling	Hotel Concierge Attendant
Participant#7	M	24	Social Work	Valet Parking Attendant
Participant#8	F	27	Public Health and Social Work	Receptionist
Participant#9	F	28	Psychological Counselling	Restaurant Servant
Participant#10	M	29	Psychological Counselling	Advertisement Assistant
Participant#11	F	24	Psychological Counselling	Marketing Assistant
Participant#12	M	25	Psychological Counselling	Security Supervisor

**Table 2:** The themes and subthemes.

Themes and Subthemes		
3.1.	Public Health, Social Work, and Psychological Counselling Services: Positions are Reserved	
3.2.	Modelling Peers	
	3.2.1.	Modelling and Referral from Classmates
	3.2.2.	Modelling and Referral from Cousins
3.3.	Lack of Career Development Skills	
	3.1.	Afraid to Start Own Centers and Non-Profit Organizations
	3.2.	Lack of Interdisciplinary and Practical Skills
